# Disease Management of Clinical Complete Responders to Neoadjuvant Chemotherapy of Muscle-Invasive Bladder Cancer: A Review of Literature

**DOI:** 10.3389/fonc.2022.816444

**Published:** 2022-04-13

**Authors:** Jie Wu, Rui-Yang Xie, Chuan-Zhen Cao, Bing-Qing Shang, Hong-Zhe Shi, Jian-Zhong Shou

**Affiliations:** Department of Urology, National Cancer Center/National Clinical Research Center for Cancer/Cancer Hospital, Chinese Academy of Medical Sciences and Peking Union Medical College, Beijing, China

**Keywords:** muscle-invasive bladder cancer, neoadjuvant chemotherapy, clinical complete response, conservative strategy, survival

## Abstract

Muscle-invasive bladder cancer (MIBC) is an aggressive disease requiring active management. Neoadjuvant chemotherapy (NAC) followed by radical cystectomy (RC) is considered the standard treatment paradigm for MIBC patients, which could result in significant perioperative mortality and morbidity, as well as the significant alteration of the quality of life (QOL). Notably, multimodal bladder-preserving treatment strategies have been recommended for highly selected patients. Pathologic complete response (pCR) after NAC is a powerful prognostic indicator of survival for patients with MIBC. Clinical complete response (cCR) is then introduced as a complementary endpoint for pCR to assess disease status preoperatively. Bladder preservation strategy for patients who achieve cCR following NAC is emerging as a new treatment concept. However, the efficiency of the conservative strategy remains controversial. In this state-of-the-art review, we discuss the advantages and limitations of cCR and the feasibility and safety of bladder preservation strategy in highly selected MIBC patients who achieve cCR following NAC. We conclude that a conservative strategy can be considered a reasonable alternative to RC in carefully selected cCR MIBC patients, leading to acceptable oncological outcomes.

## Introduction

Bladder cancer is the 12th most commonly diagnosed malignancy, with over 570,000 new cases and 210,000 deaths worldwide every year (1, 2). Bladder cancer is a biologically heterogeneous disease with various clinicopathological characteristics and outcomes. Urothelial cancer (UC) is the predominant histologic type of bladder cancer, accounting for more than 90% of the cases (3). Approximately, 25% of the tumors invade the detrusor muscle, classified as muscle-invasive bladder cancers (MIBC). The remaining are confined to the mucosa (pTa), submucosa (pT1), or carcinoma *in situ* (CIS), classified as nonmuscle-invasive bladder cancers (NMIBC) (4). Despite the high frequency of early recurrence risk, the 5-year overall survival (OS) rate for NMIBC is approximately 90%. However, the repeated tumor resections and endoscopic assessments make NMIBC a large societal health burden (5, 6).

MIBC patients have a higher propensity to experience lymph node involvement and distant metastasis. For clinically localized MIBC patients, neoadjuvant chemotherapy (NAC) followed by radical cystectomy (RC) and pelvic lymphadenectomy is considered the standard treatment choice (7). Despite the aggressive treatment strategy, up to 50% of patients with MIBC experience local relapse or distant metastasis due to disseminated micrometastases, and the 5-year overall OS rate for MIBC is approximately 50% to 60% (8, 9). Moreover, the survival benefits provided by RC should be weighed against prolonged hospitalizations, risk of perioperative mortality and morbidity, and the changes of quality of life (QOL) in long-term survivors (10, 11). Notably, multimodal bladder-preserving treatment strategies with maximal transurethral resection of bladder tumor (TURBT), radiation therapy (RT), and concurrent chemotherapy have been recommended for highly selected patients (7, 12–14).

Characterizing responders to NAC is important for patient evaluation and disease management. Previous studies have demonstrated that achieving pT0, pN0, and negative surgical margins, defined as pathologic complete response (pCR), is a powerful prognostic factor of OS (15). Clinical complete response (cCR) is then introduced as a complementary endpoint for pCR to assess disease status preoperatively, while the standardized assessments to define cCR remain heterogeneous (16, 17). Recently, conservative strategy following cCR to NAC is emerging as a new treatment concept for nonmetastatic MIBC patients. Although existing evidence reveals a disconnect between cCR and pCR, several studies have shown favorable oncological outcomes in patients achieving cCR following NAC who avoided RC (18, 19).

## Neoadjuvant Cisplatin-Based Chemotherapy in Muscle-Invasive Bladder Cancer

### History of Neoadjuvant Chemotherapy for Muscle-Invasive Bladder Cancer

The addition of combination NAC to RC was first tested in the 1980s by Scher et al. and then has been demonstrated to improve oncological outcomes of patients with nonmetastatic MIBC by several randomized phase III trials (20–22). The Nordic Cystectomy Trial I and the Nordic Cystectomy Trial II were the earliest randomized phase III trials of cisplatin-based neoadjuvant chemotherapy in MIBC, demonstrating the feasibility and safety of combining NAC before RC (23). The Southwest Oncology Group (SWOG)/Intergroup 8710 (SWOG-8710) study then provided the first level I evidence supporting the significant survival benefit with the use of the methotrexate, vinblastine, doxorubicin, and cisplatin (MVAC) regimen prior to RC, with the improvement in 5-year OS from 45% to 57% (*p* = 0.06) (21). The BA06 30894 trial enrolled 976 nonmetastatic MIBC patients exploring the efficiency of neoadjuvant cisplatin, methotrexate, and vinblastine (CMV) regimen prior to RC, considered as the largest study of NAC completed to date (22, 24). The long-term results of the BA06 30894 trial revealed that the addition of CMV improved 10-year OS from 30% to 36% (*p* = 0.037). A meta-analysis published in 2016 included 3,285 patients in 15 randomized clinical trials, showing an absolute OS benefit of 8% with cisplatin-based NAC (25). More recently, modified dose-dense (dd) MVAC was introduced in two single-arm phase II studies to overcome the toxicity of the standard-dose MVAC regimen (26, 27). ddMVAC was proven to have similar clinical efficacy with more tolerable toxicity. The median time from the initiation of the NAC regimen to surgery was shortened from 16 to 19 weeks for standard-dose MVAC to 9.7 weeks for ddMVAC.

### Trends in Utilization of Neoadjuvant Chemotherapy for Muscle-Invasive Bladder Cancer

The NAC utilization is continuously growing in the management of nonmetastatic MIBC after the SWOG-8710 trial, demonstrating the improved OS with the use of preoperative chemotherapy (21, 28). Multidisciplinary treatment (MDT) for comprehensive management of MIBC also allows improvement of NAC strategies and rising NAC utilization (29). Reardon et al. and McFerrin et al. performed a retrospective analysis based on the National Cancer Database to determine trends in NAC used within the United States population. The results revealed that the NAC utilization increased from 10.1% in 2006 to 20.8% in 2010 and reached 32.3% in 2015 (30, 31). John et al. provided an overview of nonmetastatic MIBC patients diagnosed in England, retrospectively utilizing the National Cancer Registration and Analysis Service database (32). They found that the NAC utilization had reached 37% in England in 2016. A retrospective analysis based on the four academic hospitals in Japan also demonstrated that NAC utilization increased from 9.7% in 2004 to 96% in 2016 (33). The underutilization of NAC is mainly attributed to poor performance status, impaired renal function, and severe comorbidities. Galsky et al. proposed the first cisplatin ineligibility criteria based on renal function (34). Notably, nearly 50% of MIBC patients assigned to receive RC are defined as cisplatin ineligible and excluded from NAC (35, 36). Thus, on account of the 30%–40% overall utilization rate, the NAC has been commonly applied in cisplatin-eligible MIBC patients.

### Role of Neoadjuvant Immunotherapy

The immune checkpoint inhibitors (ICIs) have deeply changed the therapeutic paradigm of BC. Inhibition of PD-1/PD-L1 has been proved for locally advanced and metastatic UC as second-line treatment. Pembrolizumab and atezolizumab have been recommended in cisplatin-ineligible patients with high PD-L1 expression as first-line therapy by the US Food and Drug Administration (FDA) and the European Medicines Agency (EMA), and further in patients who are not eligible for platinum-containing treatment regardless of PD-L1 status only by the FDA (37). Five ICIs (pembrolizumab, atezolizumab, nivolumab, avelumab, and durvalumab) have been proved for metastatic BC progressing after platinum-based chemotherapy as second-line treatment by the FDA, while only three ICIs (pembrolizumab, atezolizumab, and nivolumab) have been proved by the EMA (38). Notably, the recommendation of pembrolizumab after platinum failure is independent of PD-L1 expression levels, and avelumab has been recommended as a maintenance treatment following first-line platinum-containing chemotherapy (7, 39–42). The role of ICIs in the neoadjuvant setting has also been explored. PURE-01 and ABACUS are two single-agent neoadjuvant immunotherapy trials assessing the efficacy and safety of pembrolizumab and atezolizumab monotherapies in patients with MIBC, respectively (43, 44). In PURE-01, 42% of patients achieved the pCR, and the 1- and 2-year event-free survival (EFS) was 84.5% and 71.7%, respectively (43, 45). In ABACUS, 31% patients achieved the pCR, and 2-year RFS and 2-year OS were 77% and 82%, respectively (44). NABUCCO is a single-arm neoadjuvant combination immunotherapy trial testing the feasibility of preoperative ipilimumab plus nivolumab and the pCR rate was reported as 46% (46). BLASST-1 assessed the efficiency of neoadjuvant combination of immunotherapy and chemotherapy. The pCR rate of patients receiving neoadjuvant nivolumab, gemcitabine, and cisplatin was reported as 49% (47). The emerging results from several clinical trials exploring the role of neoadjuvant immunotherapy are promising; however, further long-term survival data are needed to demonstrate the survival benefit of neoadjuvant immunotherapy.

## Clinical Complete Response After Neoadjuvant Chemotherapy in Muscle-Invasive Bladder Cancer

### Pathologic Complete Response Is a Powerful Prognostic Indicator of Survival for Patients Receiving Neoadjuvant Chemotherapy

NAC followed by RC has been shown to improve the outcome of MIBC patients compared with surgery alone. However, the benefit of NAC is restricted to only a subgroup of patients, and the stage of disease after RC is the key prognostic factor of MIBC patients (48). Randomized clinical trials and meta-analyses further demonstrated that pCR after NAC is a powerful prognostic indicator of survival for patients with MIBC. The SWOG-8710 study revealed that the 5-year OS of pCR patients was 85%, significantly higher than the no-pCR group (21). A meta-analysis published in *European Urology* in 2013 also confirmed that pCR is correlated with a 26% lower absolute risk of mortality and a 51% absolute lower risk of recurrence compared with pathologic residual disease (49). Due to its ability to predict clinical benefits, pCR has been used as a surrogate endpoint in clinical trials to explore novel NAC regimens. Approximately, 40% of MIBC patients are downstaged to pT0N0M0 disease following NAC, indicating the chance of a conditional long-term survival (50, 51).

### Clinical Complete Response After Neoadjuvant Chemotherapy in Muscle-Invasive Bladder Cancer

However, patients with pCR disease can only be identified after RC. In that case, cCR before RC has been proposed as an intriguing intermediate endpoint to assess disease status preoperatively. Reliable identification of clinical responders would definitely improve the treatment strategies of MIBC and offer a QOL advantage and avoid attendant morbidity and mortality of RC in highly selected patients (16, 17). Unfortunately, the standardized assessments to define cCR remain highly heterogeneous. Common restaging evaluation contains repeat cystoscopy under anesthesia with biopsy, TURBT down to muscularis propria, cross-sectional imaging, and urine cytology (17, 52). The main concern of cCR as a predictor of treatment efficacy is that the post-NAC clinical restaging cannot identify patients with residual invasive disease accurately. Kukreja et al. evaluated the correlation between cT0 from maximal TURBT and pT0 on final RC specimens in 157 RC patients identified as stage cT0. The result revealed that only 56 (35.7%) patients were pT0 on final RC pathology. Notably, lymph node involvement at RC was found in 20 (12.7%) of the patients despite cross-sectional imaging confirming cN0 (18). Becker et al. assessed the accuracy of clinical restaging and found that among 53 patients identified as cT0 patients based on restaging TUR, only 25 (47%) were ultimately pT0 based on the pathologic outcomes (53). This existing evidence reveals the inaccuracy of the current assessment of cCR and highlights the importance to construct standardized assessments of cCR. However, among 17 TUR-confirmed cT0 patients with visually negative cystoscopy, 10 (59%) were ultimately pT0, indicating the improved accuracy with additional staging method (53).

Over the last decade, several novel imaging techniques have been explored to assess the response of MIBC patients to NAC, including positron emission tomography and computed tomography (FDG-PET/CT) scans, multiparametric MRI, and dynamic contrast-enhanced MRI (54–56). Urinary cytology plays a significant role in the detection and surveillance of bladder cancer, and detection of soluble proteins and omics biomarkers have been further explored, such as the detection of nuclear matrix proteins and tests of DNA methylation (57, 58). Recently, liquid biopsy based on urine tumor DNA has also been demonstrated to be a highly sensitive technique in the detection of minimal residual disease (MRD) (59). These tests could be reliable complements to urinary cytology with high sensitivity. Moreover, several enhanced endoscopic techniques including fluorescence cystoscopy, optical coherence tomography, confocal laser endomicroscopy, and narrow-band imaging have emerged to improve the diagnostic accuracy of BC (60–63). Integration of advanced imaging, endoscopic techniques, and precise MRD detection approaches represents potential complements to refine assessments of cCR following NAC.

## Disease Management of Clinical Complete Responders to Neoadjuvant Cisplatin-Based Chemotherapy of Muscle-Invasive Bladder Cancer

### Treatment of Muscle-Invasive Bladder Cancer

NAC followed by RC is the current standard of care of MIBC patients when surgery can be tolerated, supported by the National Comprehensive Cancer Network (NCCN) guidelines and the updated European Association of Urology (EAU) guidelines as a category 1 recommendation (7, 51). However, RC is a major surgery associated with significant postoperative morbidity and mortality. The 30- and 90-day mortality was reported to be as high as 2.7% and 2.3%–7.2%, respectively (10, 64). Also, the risk of early complications was estimated to be 28%–58% (8, 65). Thus, 47%–51% of MIBC patients did not choose a curative-intent treatment strategy (66). Multimodality bladder-preserving strategies have emerged with continued refinements over the past two decades. Maximal TURBT and concurrent chemotherapy and RT constitute the main structure of trimodal therapy (TMT), which has been extensively studied and widely used in noncystectomy candidates (67, 68). A pooled analysis of radiation therapy oncology group (RTOG) protocols demonstrated the efficiency of combined-modality therapy, with a 5-year OS and 5-year disease-specific survival of 57% and 71%, respectively (12). A meta-analysis included 11 studies comparing the effectiveness of TMT versus RC, and the results showed no statistical difference in OS (HR: 1.06; 95% CI: 0.85–1.31) but poorer CSS (HR: 1.23; 95% CI: 1.04–1.46) for patients who received TMT (69). Moreover, several ongoing studies about the conservative strategy in MIBC patients are being actively investigated, including HCRN GU 16-257, NCT02621151, IMMUNOPRESERVE-SOGUG, NCT03747419, NCT03775265, NCT03617913, and NCT03697850 (51, 70).

### Conservative Strategy Following Complete Clinical Response to Neoadjuvant Chemotherapy of Muscle-Invasive Bladder Cancer

It remains highly controversial whether conservative strategy in MIBC patients following cCR to NAC is safe or associated with a high risk of mortality, and most patients underwent RC directly without a restaging evaluation. However, considering the substantial perioperative mortality and morbidity, as well as the significant alteration of QOL due to RC, bladder preservation therapy warrants exploration. Therefore, the efficiency of bladder preservation strategies for patients who achieve cCR following NAC has been preliminarily explored **(** **Table 1****)**. Harry reported the outcomes of 63 nonmetastatic MIBC patients that initially refused RC after achieving cCR following NAC. The results revealed that during the 86 months of median follow-up, 64% of the patients survived, and 54% of the survivors had an intact functioning bladder (71). Meyer et al. reported the clinical course of 32 MIBC patients that achieved cCR following NAC. The 5-year cancer-specific survival (CSS) of the 25 patients who opted for bladder preservation was 88% (72). The study performed by Robins et al. reported a 5-year CSS of 87% for 41 MIBC patients that declined RC after achieving cCR following NAC (73). Furthermore, a retrospective multi-institutional study carried out by Mazza et al. reported contemporary outcomes of 148 nonmetastatic MIBC patients who opted for conservative management after achieving cCR following radical TURBT then by NAC. At a median surveillance of 55 months, the 5-year OS and CSS of the entire cohort were 86% and 90%, respectively, with 82% of survivors retaining their bladder (19). A more recent study carried out by Onishi et al. reported the clinical outcomes of 58 patients who desire bladder preservation after receiving noninvasive downstaging following NAC, which was defined as ≤T1 disease at first-TURBT after NAC and cCR on second-TURBT. During a median follow-up of 40 months, the 5-year OS of noninvasive downstaging patients was 89% (74). Notably, four patients receiving neoadjuvant pembrolizumab refused RC after achieving cCR in PURE-01, and none of them experienced recurrence in a median follow-up of 10 months, indicating the potential feasibility of the conservative approach after neoadjuvant immunotherapy (45). Besides post-NAC surveillance, the efficiency of chemoradiotherapy (CRT) has also been explored in MIBC patients who received NAC. A retrospective cohort study conducted by Jiang et al. enrolled 57 MIBC patients who received CRT following NAC. The 2-year OS was 74%, and cCR was identified as an independent risk factor (75). In a phase II study carried out by Shi et al. with the purpose to assess the efficiency of NAC-guided bladder-sparing treatment in MIBC patients, 6 patients who achieved cCR underwent TURBT plus concurrent CRT and all survived with a functional bladder in a median follow-up of 44.6 months (76). These findings demonstrated the feasibility and safety of bladder preservation strategy in highly selected cCR MIBC patients.

**Table 1:** Studies of conservative strategy following complete clinical response to neoadjuvant chemotherapy of muscle-invasive bladder cancer.

Study	No. of patient who achieved cCR	Median follow-up	Recurrence rate	Outcome	References
Harry (2008)	63	86	30%	5-year OS: 64%	(71)
Meyer (2014)	32	NA	52%	5-year CSS: 88%	(72)
Robins (2017)	48	35	46%	5-year OS: 87%	(73)
Mazza (2018)	148	55	48%	5-year OS: 86%	(19)
				5-year CSS: 90%	
Onishi (2021)	58	40	36%	5-year OS: 89%	(74)

cCR, complete clinical response; OS, overall survival; CSS, cancer-specific survival.

## Discussion

Avoiding RC for patients who achieved cCR following NAC has been explored to some extent. However, bladder-preservation treatment may add the risk of tumor recurrence by forgoing immediate RC. The main concern of any alternatives to RC has been the possibility that endoscopically cCR could not precisely identify residual disease. Notably, despite the high survival rate in MIBC patients who opted for conservative management after achieving cCR following NAC, the intravesical recurrence rate of these patients could reach 30% to 50% **(** **Table 1****)**. Recurrences may be due to either undetected residual disease or new primary tumors, and the former assumption indicated the suboptimal assessments of current clinical response to NAC. However, most of the recurrence cases are nonmuscle invasive and do not definitely correlate with poor outcomes. Among 71 of the 148 patients (48%) who experienced intravesical recurrence in the study conducted by Mazza et al., 55 (77%) were nonmuscle invasive and only 11 (15%) eventually died due to cancer (19). Bladder cancer is more than a malignancy, and referring to disease management strategy, the important underlying point is clinical benefit and QOL. Conservative strategy provides MIBC patients who achieving cCR following NAC an opportunity for long-term survival with a functioning bladder. Although current assessments of cCR are deficient, the stability of cCR to predict survival will significantly impact disease management of MIBC. After all, the reference standard pCR is merely another surrogate endpoint for OS.

Selection criteria of conservative strategy are the key to the oncologic outcomes of patients, as MIBC represents a group of heterogeneous tumors. The initial inclusion criteria are the nonmetastatic MIBC patients who were recommended to undergo RC while elected bladder-preserving treatment strategies after achieving cCR following NAC. Patients were not preselected for the bladder-preserving protocol in the studies performed by Harry, Meyer et al., and Robins et al. (71–73). However, Harry found that the larger size (>5 cm) and multiple invasive tumors were independently associated with poorer OS (71). Notably, in the study carried out by Mazza et al., the patients tended to have solitary muscle-invasive lesions and low volume (<5 cm) (19). Onishi et al. also excluded patients with a tumor larger than 10 cm (74). Thus, nonmetastatic MIBC patients with low volume and solitary muscle-invasive lesions may better respond to conservative strategy after achieving cCR following NAC. However, the bladder-preservation protocols are needed to be optimized to better identify proper patients who can benefit from this conservative approach.

Pooling the available evidence, NAC followed by RC is still the current standard of care of MIBC patients, while for patients who achieved cCR following NAC, the conservative strategy could also lead to acceptable oncological outcomes. Thus, for cCR patients with a desire to preserve their bladder and willing to undergo surveillance, the conservative strategy could be a reasonable choice.

## Conclusion

MIBC is an aggressive disease requiring active management. Cisplatin-based NAC followed by RC is the current standard treatment of clinically localized MIBC patients. Despite the high intravesical recurrence rate, conservative strategy can be considered a reasonable alternative to RC in carefully selected cCR MIBC patients followed by TURBT and NAC, leading to acceptable oncological outcomes and better QOL. Looking to the future, a standardized definition of cCR is sorely needed to better assess disease status preoperatively, and well-designed randomized phase III trials are needed to validate the efficacy and safety of conservative strategy.

## Author Contributions

JW and RY-X conceived the idea and was a major contributor in manuscript writing. CZ-C and BQ-S was involved in the manuscript writing. HZ-S and JZ-S designed the research. All authors listed have made a substantial, direct, and intellectual contribution to the work and approved it for publication.

## Conflict of Interest

The authors declare that the research was conducted in the absence of any commercial or financial relationships that could be construed as a potential conflict of interest.

## Publisher’s Note

All claims expressed in this article are solely those of the authors and do not necessarily represent those of their affiliated organizations, or those of the publisher, the editors and the reviewers. Any product that may be evaluated in this article, or claim that may be made by its manufacturer, is not guaranteed or endorsed by the publisher.
